# Clinical Outcomes and Complications of the Suzuki Frame in Digital Fractures: A Systematic Review and Meta-Analysis

**DOI:** 10.7759/cureus.101916

**Published:** 2026-01-20

**Authors:** Juber Ahmed, Kouther Mohsin, Mohammed R Rahman, Osama Embaby, Saad Elashry

**Affiliations:** 1 Trauma and Orthopaedics, Sandwell and West Birmingham Hospitals NHS Trust, Birmingham, GBR; 2 Emergency Medicine, University Hospitals of North Midlands NHS Trust, Birmingham, GBR; 3 Orthopaedics, Mansoura University Hospital, Birmingham, GBR

**Keywords:** dynamic external fixation, hand injuries, phalangeal fractures, pins and rubber traction system, suzuki frame

## Abstract

Intraarticular fractures of the phalanges and thumb base are challenging to manage due to the risks of stiffness and loss of motion. The Suzuki pins and rubbers traction system provides dynamic external fixation that maintains reduction while allowing early mobilisation. No prior systematic review and meta-analysis (SRMA) has comprehensively evaluated its outcomes. This study aimed to summarise radiographic union, functional recovery and complication rates to inform clinical decision-making. In accordance with Preferred Reporting Items for Systematic Reviews and Meta-analyses (PRISMA) 2020 guidelines, MEDLINE and Embase were searched from 1994 to October 2025. Eligible studies included ≥5 patients with intraarticular phalangeal or thumb fractures managed using the Suzuki frame. Primary outcomes included range of motion (ROM), union rate and grip strength. Data extraction was performed in Microsoft Excel (Microsoft Corporation, Redmond, WA, US), screening in Rayyan and analysis in OpenMetaAnalyst, using fixed or random effect models with 95% CIs and Cochran’s Q and I² tests. Twelve studies (183 patients, 185 fractures) were included in the analysis. The pooled union rate was 97.8% (95% CI 0.95 - 1.00, p<0.001). Functional recovery was favourable; pooled proximal interphalangeal joint (PIPJ) range of motion (RoM) was 89.6 degrees (95% CI 88.4 - 90.7, p<0.001). Distal interphalangeal joint (DIPJ) ROM was 66.0 degrees (95% CI 64.6 - 67.4, p<0.001), and grip strength was 81.2% (95% CI 78.3 - 84.0, p<0.001). Pin site infection rate was 10.3% (95% CI 0.0 - 0.2, p<0.001), and joint space narrowing was 5% (95% CI 0.0 - 0.1, p<0.05). Post-traumatic arthritis occurred in 2.7%. Pain was minimal (visual analogue scale (VAS) <2). The Suzuki traction system achieves high union, satisfactory ROM and low complication rates in intraarticular digital fractures. Maintaining traction for four to six weeks optimises healing. Its minimally invasive design enables early motion and supports functional recovery and quality of life.

## Introduction and background

Fractures of the hand account for 20% of all fracture presentations to emergency departments in the UK [1]. Management options vary depending on the location, pattern and stability of the fracture. Conservative treatment, such as splinting, may be appropriate in cases where the fracture is stable, minimally displaced and unlikely to compromise long-term function [2].

Interphalangeal joint fractures pose clinical challenges, as they can result in stiffness, non-union and long-term functional impairment. Consequently, surgical intervention is often required to restore alignment, appearance, and ultimately function. Poorly managed phalangeal fractures may significantly compromise dexterity and quality of life [3].

Many methods are currently employed to manage these complex fractures. Management options for stable fractures include conservative immobilisation and splinting. For unstable and comminuted fractures, management options include open reduction and internal fixation (ORIF), static external fixation and dynamic digital external fixation (DDEF). ORIF offers rigid stabilisation but requires soft tissue dissection, which may increase the risk of adhesions and postoperative stiffness. Static external fixation provides stability but restricts motion. The dynamic external fixation technique was developed to provide stable traction whilst permitting mobilisation.

The pins and rubbers traction system, first described by Suzuki et al. in 1994, is now widely referred to as the Suzuki frame [4]. The device features a simple, compact design comprising Kirschner (K) wires connected by elastic bands to create dynamic external traction. The first K-wire serves as an axial traction pin, inserted through the injured phalanx, while the second K-wire functions as a hook pin, positioned through either the injured or adjacent phalanx, depending on fracture configuration. Rubber bands connect the pins, forming a tensioned traction system. In some cases, a third K-wire may be added as a reduction pin, typically inserted into the base of the middle phalanx to enhance alignment. By applying controlled dynamic traction across the joint, the device enables closed reduction of intraarticular fracture fragments via capsuloligamentotaxis while simultaneously allowing early controlled range of motion.

Over the past three decades, numerous small case series and observational studies have reported promising outcomes regarding the use of the Suzuki frame in the management of intraarticular fractures of the proximal, middle and distal phalanges, as well as the base of thumb. These studies consistently describe good fracture healing and functional recovery, though less favourable outcomes, such as pin-site infection, post-traumatic arthritis, extensor lag and osteomyelitis, have also been reported.

The evidence base, however, is largely limited to small single-arm, retrospective case series with varying outcome reports. The lack of randomised controlled trials and the absence of systematic synthesis of the available data make it difficult for surgeons to draw definitive conclusions regarding the comparative efficacy and safety of the Suzuki traction system compared with other treatment options. A systematic review and meta-analysis is required to consolidate available data, quantify union and functional outcomes and better define the role of the Suzuki frame in the management of complex intra-articular digital fractures.

## Review

Methods

This systematic review and meta-analysis were performed in accordance with the Preferred Reporting Items for Systematic Reviews and Meta-analyses (PRISMA) guidelines [5].

*Eligibility Criteria*

Studies were included if they involved patients with base of thumb proximal phalanx fractures or with proximal or distal interphalangeal joint fractures treated using the Suzuki frame. Eligible study designs included randomised controlled trials, cohort studies, and case series with a minimum of five patients. There were no restrictions on the year of publication. Studies were excluded if they did not use the Suzuki frame, used a significantly modified technique, lacked clear outcome measures, consisted of fewer than five patients or were based on animal or cadaveric studies. Unpublished data and non-English language publications were also excluded.

Search Strategy

The electronic databases MEDLINE and Embase were searched. Search terms included “Suzuki frame,” “Suzuki device,” “pins and rubber device,” “phal*,” “interphalangeal,” “fracture,” “thumb,” and “injury.” Boolean operators (AND, OR) were used to optimise results. The reference lists of relevant articles were also screened to find further eligible studies. The last search was performed in October 2025. The searches were performed independently by two reviewers (JA, KM).

Study Selection Process

Initial screening of titles and abstracts of all studies retrieved from the literature searches was independently assessed by two authors (JA, KM) using the Rayyan platform [6]. Duplicates were removed prior to screening. All articles that met the eligibility criteria were selected, and the full texts of the articles were reviewed. Any disagreements between reviewers were resolved through discussion.

Data Extraction

Data was extracted using a Microsoft Excel version 2509 sheet adapted from Cochrane's data collection form for intervention [7,8]. The extraction form was piloted on a small number of studies to ensure consistency before using the full data set. Data extracted included study characteristics (author, year, study design, sample size), patient demographics (age, sex distribution, affected digit, fracture patterns), treatment characteristics (duration of traction and follow-up period) and clinical outcomes (radiographic fracture union, range of motion (ROM) at the metacarpophalangeal joint (MCPJ), Proximal Interphalangeal Joint (PIPJ), Distal Interphalangeal Joint (DIPJ), grip strength, pain, QuickDash, infections, and joint space narrowing). Two reviewers (JA, KM) independently extracted all data and cross-checked inputs, with any ambiguity resolved by discussion.

Risk of Bias Assessment

The risk of bias was evaluated using the Newcastle Ottawa scale (NOS) for observational studies [9]. NOS assesses studies across three domains: selection (maximum four stars), comparability (maximum two stars) and outcome (maximum three stars), with the maximum score being nine. Higher scores were interpreted as indicating lower risk of bias and stronger methodological quality. The following score thresholds were used: 1-3 = high risk, 4-6 = moderate risk, and 7-9 = low risk of bias.

Statistical Analysis

For continuous outcomes, the mean, standard deviation, and 95% confidence intervals were extracted or calculated. For dichotomous outcomes, the event pooled proportions with 95% CI were used. Odds ratios were not calculated, as no study reported control groups. Meta-analyses were performed when at least five studies reported the same outcome. Analyses were conducted using OpenMetaAnalyst Version 3.1 [10]. Heterogeneity between studies was assessed with Cochran’s Q test and quantified using the I² statistic. An I² value <50% was considered to represent low heterogeneity, while values ≥ 50% considered substantial heterogeneity. A fixed effects model was used where heterogeneity was low, and a random effects model was used where heterogeneity was high.

Results

Literature Search Results

**Figure 1:**
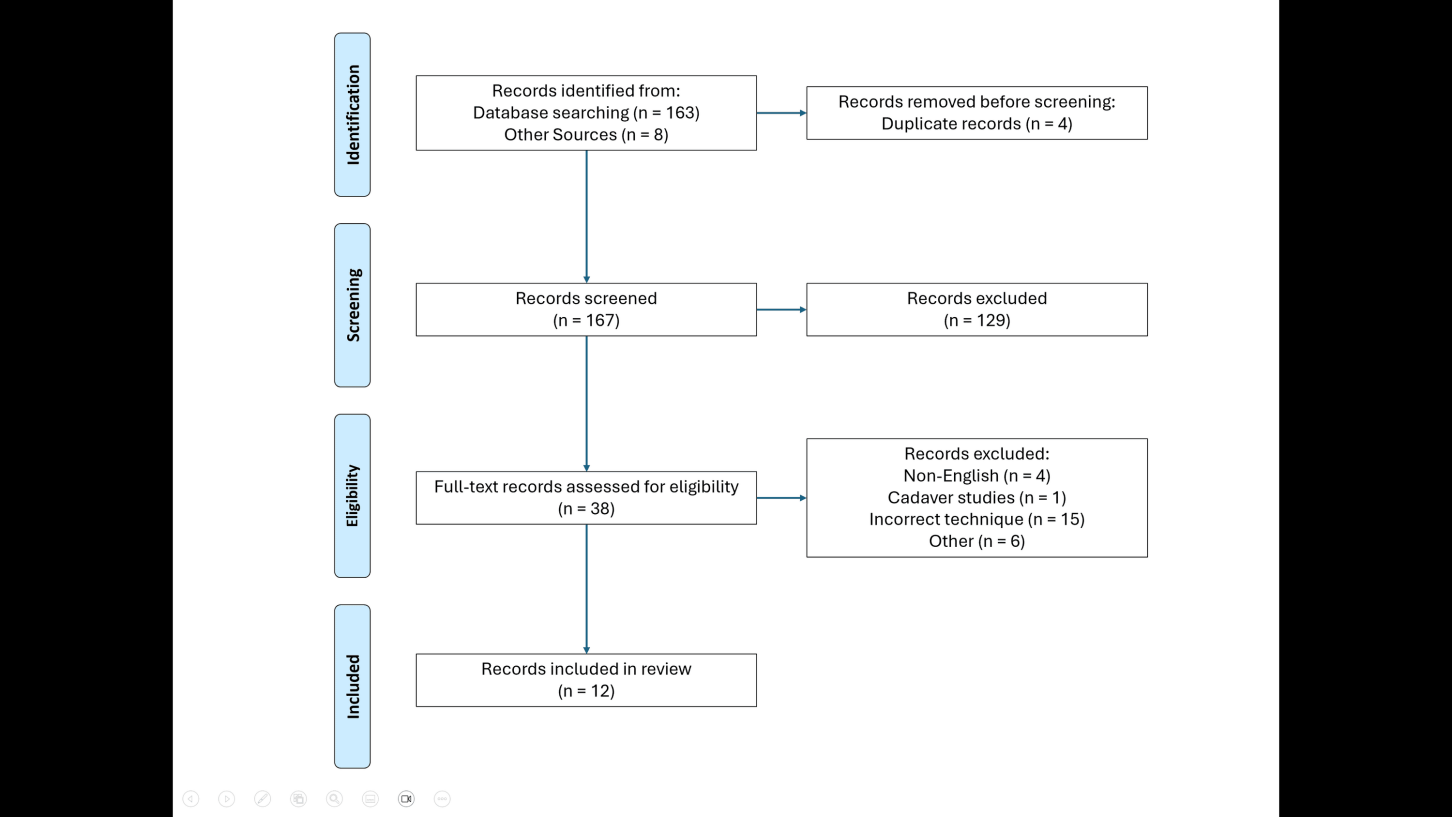
PRISMA 2020 flow diagram illustrating the study selection process, from the initial search to the final inclusion of studies PRISMA: Preferred Reporting Items for Systematic Reviews and Meta-Analyses

A total of 171 studies were identified through database and other source searches. After the removal of 4 duplicates, 167 unique studies remained for screening. A total of 129 studies were excluded based on title and abstract. Thirty-eight full-text articles were assessed for eligibility, and 26 were excluded. Twelve studies met the eligibility criteria and were included for the final analysis [4,11-21]. The study selection process is illustrated in the PRISMA flow diagram (Figure 1).

Study Characteristics

A total of 12 observational studies were included in this review, all published between 1994 and 2024 (Table 1). Collectively, these studies comprised 183 patients with 185 phalangeal fractures managed using the pins and rubbers traction system. All studies were retrospective case series, with two studies reporting consecutive series [14,17].

**Table 1:** Overview of key study characteristics from the 12 included studies evaluating the use of the Suzuki pins and rubbers traction system in interphalangeal fractures

						Affected digit					Fracture pattern					
Author	Year	Study type	Number of Patients, n	Number of Fractures, n	Age, Mean (SD), years	Thumb	Index	Middle	Ring	Little	Volar	Dorsal	Pilon/Comminuted	Other	Duration of Traction, Mean (SD), days	Follow-up Duration, Mean (SD), months
Suzuki et al. [4]	1994	Retrospective Case Series	6	6	41.8 (17.8)	-	2	1	2	1	-	-	-	-	33.8 (1.0)	13.5 (5.9)
De Soras et al. [11]	1997	Retrospective Case Series	11	11	36.0 (1.7)	-	-	-	-	-	3	-	-	8	NR	9.7 (NR)
De Smet and Fabry [12]	1998	Retrospective Case Series	5	5	42.0 (15.1)	-	1	2	2	-	-	-	-	-	NR	NR
Duteille et al. [13]	2003	Retrospective Case Series	16	16	28.2 (16.2)	0	1	4	10	5	-	-	9	-	25.2 (8.3)	18.0 (NR)
Majumder et al. [14]	2003	Retrospective Consecutive Case Series	14	14	28.0 (12.0)	-	-	-	-	-	8	3	3	0	35.0 (6.2)	20.0 (6.2)
Keramidas and Miller [15]	2005	Retrospective Case Series	5	5	35.4 (9.5)	5	-	-	-	-	-	-	5	-	28.0 (NR)	23.6 (6.9)
Keramidas et al. [16]	2007	Retrospective Case Series	11	11	30 (10.7)	-	-	6	-	-	-	-	6	5	28.0 (NR)	18.0 (NR)
Debus et al. [17]	2010	Single-Centre Retrospective Consecutive Case Series	15	15	39.5 (12.1)	-	-	4	5	6	8	3	4	0	37.8 (16.1)	53 (19.6)
Finsen [18]	2010	Retrospective Case Series	18	19	50.6 (16.0)	-	-	6	6	6	10	1	7	2	32.5 (10.4)	57.7 (27.0)
Kiral et al. [19]	2014	Retrospective Case Series	33	33	23.0 (10.1)	7	7	4	8	7	5	2	22	4	NR	24 (13.7)
Nanno et al. [20]	2019	Retrospective Case Series	39	39	46.0 (14.1)	0	6	9	16	8	-	-	5	-	44.8 (11.4)	8.9 (NR)
Kaplan et al. [21]	2024	Retrospective Case Series	10	11	31.1 (14.3)	-	3	2	3	2	-	-	4	-	36.0 (2.9)	13.2 (1.6)

The weighted mean patient age across studies was 35.5 years, ranging from 23 to 50.8 years, with most participants in the third to fifth decades of life. Younger cohorts presented more frequently with complex or comminuted injuries [19], reflecting high-energy mechanisms, whereas older groups predominantly included dorsal fracture injuries [18]. There was a marked male predominance with 155 males (84.5%) and 28 females (15.5%) reported.

The number of cases per study ranged from five to 39. The ring (n=52), middle (n=38) and little (n = 35) fingers were most frequently affected, followed by the index (n = 20) and thumb (n = 12) digits. The predominant fracture pattern was pilon or comminuted (n = 65), followed by volar (n = 34) and dorsal (n = 9). Other (n = 19) fracture types were also reported.

The mean duration of traction ranged from 28 to 45 days. The mean follow-up period ranged from nine to 58 months, with the longest follow-up being 116 months [18].

Primary Outcomes

ROM MCPJ:* *ROM at the MCPJ was reported by three studies, ranging from 0 to 100 degrees, and mean values from 55 to 74 degrees, indicating substantial variability. Due to sparse data reporting, quantitative pooling was not possible.

**Figure 2:**
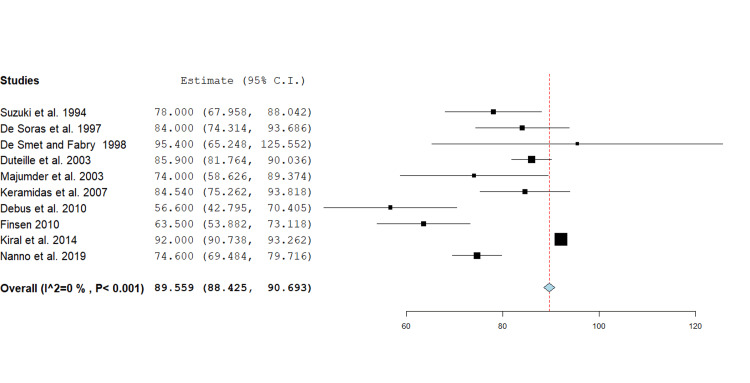
Forest plot showing mean difference analysis of range of motion at the proximal interphalangeal joint following phalangeal fracture management using the Suzuki traction system. The pooled mean across 10 studies was 89.6 degrees (95% CI 88.4 - 90.7, p<0.001). Source: [4,11-14,16-20]

ROM PIPJ: ROM at the PIPJ was reported by 10 studies, comprising 169 fractures, with a range from 0 to 130 degrees [4,11-14,16-20]. The pooled mean ROM at the PIPJ was 89.6 degrees (95% CI 88.4 - 90.7, p<0.001) with individual estimates ranging from 56.6 to 95.4 degrees and most values clustering between 70 and 90 degrees. Standard deviations, where available, ranged widely (8.4-34.4), reflecting clinical variability. A forest plot was generated using a fixed effect inverse variance model for mean differences (Figure 2).

**Figure 3:**
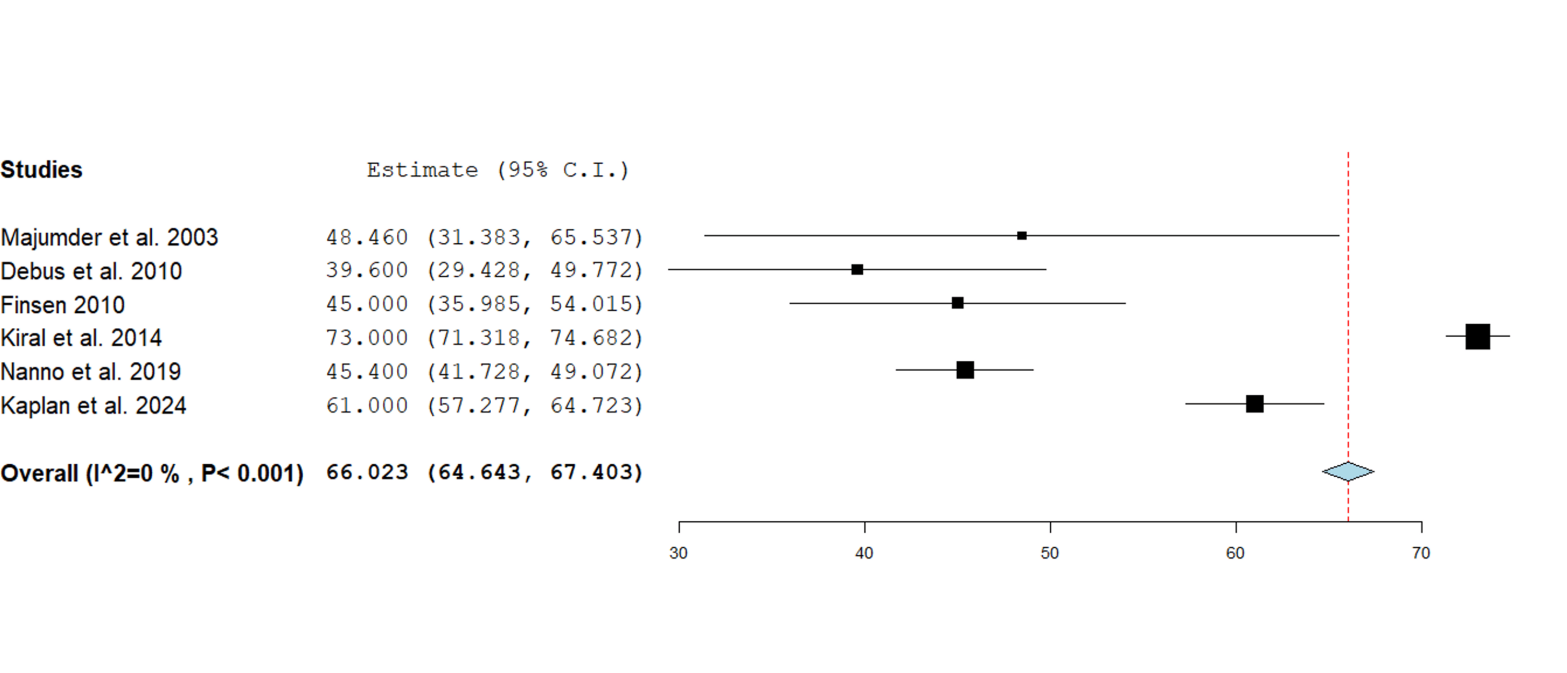
Forest plot showing mean difference analysis of range of motion at the distal interphalangeal joint following phalangeal fracture management using the Suzuki traction system. The pooled mean across six studies was 66.0 degrees (95% CI 64.6 - 67.4, p<0.001). Source: [14,17-21]

ROM DIPJ: ROM at the DIPJ was reported by six studies, comprising 131 fractures, with a range from 5 to 90 degrees [14,17-21]. The pooled mean ROM DIPJ was 66.0 degrees (95% CI 64.6 - 67.4, p<0.001) with individual estimates ranging from 39.6 to 73 degrees. The best mean result of 73 degrees was reported by Kiral et al. A forest plot was generated using a fixed effect inverse variance model for mean differences (Figure 3).

Extension lag: Extension lag was reported by two studies [17,21]. The mean PIPJ extension lag ranged from -9.6 (hyperextension) to -9.9 degrees (flexion deficit). The mean DIPJ extension lag ranged from -10 to -7.3 degrees. Due to sparse data reporting, quantitative pooling was not possible.

**Figure 4:**
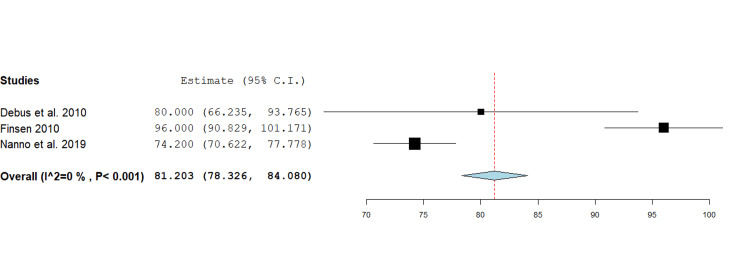
Forest plot showing mean difference analysis of grip strength following phalangeal fracture management using the Suzuki traction system. The pooled mean across three studies was 81.2% (95% CI 78.3 – 84.0, p<0.001). Source: [17,18,20]

Grip strength: Grip strength was reported by three studies, comprising 73 fractures, with a range from 23 to 118% [17,18,20]. The pooled mean grip strength was 81.2% (95% CI 78.3 - 84.0, p<0.001), with individual estimates ranging from 74.2 to 96%. A forest plot was generated using a fixed effect inverse variance model for mean differences (Figure 4).

QuickDASH:* *QuickDASH scores were reported by three studies, ranging from 0 to 48%, with reported means or medians between 2.0 and 3.2%, indicating overall excellent functional recovery and minimal long-term disability [18,20,21]. Due to sparse data reporting, quantitative pooling was not possible.

Secondary Outcomes

**Figure 5:**
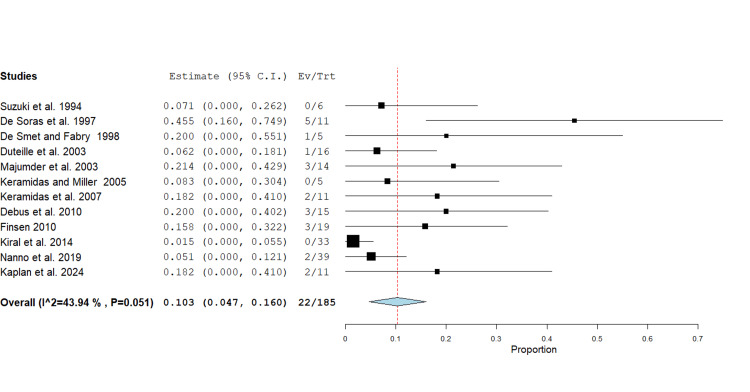
Forest plot showing proportional analysis of pinsite infections following phalangeal fracture management using the Suzuki traction system. The pooled mean across 12 studies was 10.3% (95% CI 0.0 - 0.2, p<0.001). Source: [4,11-21]

Pinsite infection:* *Twenty-two pinsite infections were reported by 12 studies, comprising 185 fractures, with a range from 0 to 5 (0-45%) [4,11-21]. The pooled pinsite infection rate was 10.3% (95% CI 0.0 - 0.2, p<0.001). Heterogeneity was moderate (I² = 43.9%). Most studies reported low rates of infection <25%, with one study reporting a higher proportion [11]. A forest plot was generated using a random effects model for mean differences (Figure 5).

**Figure 6:**
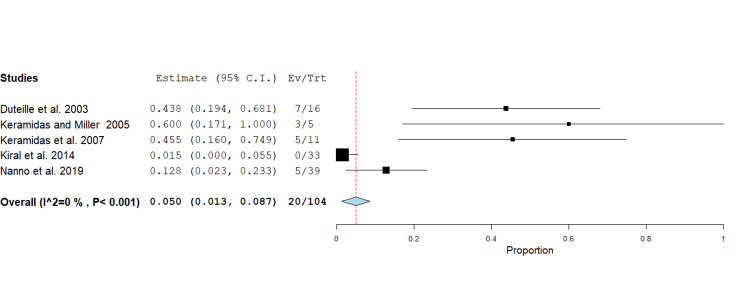
Forest plot showing proportional analysis of joint space narrowing following phalangeal fracture management using the Suzuki traction system. The pooled mean across five studies was 5% (95% CI 0.0 - 0.1, p<0.05). Source: [13,15,16,19,20]

Joint space narrowing: Twenty cases of joint space narrowing were reported by five studies, comprising 104 fractures, with a range from zero to seven cases [13,15,16,19,20]. The pooled mean rate of joint space narrowing was 5% (95% CI 0.0 - 0.1, p<0.05). In two studies, there appears to be a higher frequency of joint space narrowing in younger cohorts (mean ages 28-35 years) [13,16]. Joint space narrowing was most frequently reported in studies involving the middle, ring, and little fingers. A higher percentage of joint space narrowing was also reported in thumb injuries; however, the sample size was small [15]. A forest plot was generated using a fixed effect inverse variance model for mean differences (Figure 6).

Arthritis: Five (2.6%) cases of post-traumatic arthritis were reported by one study [20]. Due to sparse data reporting, quantitative pooling was not possible.

Pain VAS: Five studies reported Pain VAS scores [12,14,15,20,21]. Pain levels were very low across studies, with mean reported scores of less than two. Due to sparse data reporting, quantitative pooling was not possible.

**Figure 7:**
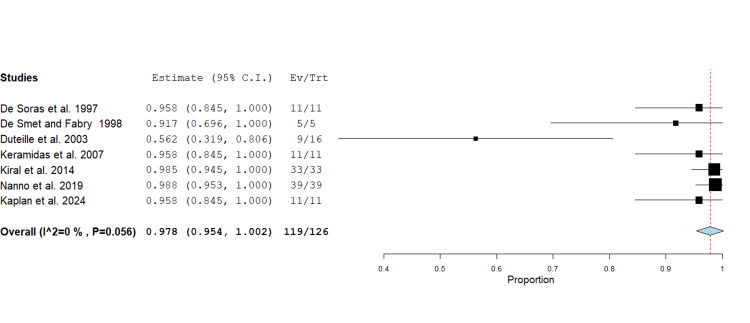
Forest plot showing proportional analysis of radiographic fracture union following phalangeal fracture management using the Suzuki traction system. The pooled mean across 7 studies was 97.8% (95% CI 0.95 - 1.00, p<0.001). Source: [11-13,16,19-21]

Radiographic Fracture Union: A total of 119 radiographic fracture unions were reported by 7 studies, comprising 126 fractures, with a range from 56.3 to 100% [11-13,16,19-21]. The pooled radiographic union rate was 97.8% (95% CI 0.95 - 1.00, p<0.001). The lowest union rate was 56.3% [13]. A forest plot was generated using a fixed effect inverse variance model for mean differences (Figure 7).

Other Reported Complications

Across the studies, several less commonly reported complications were documented in addition to the primary and secondary outcomes. Joint contractures were the most frequent of these, reported in 27 cases across 3 studies [14,19,20]. Residual oedema or persistent swelling was reported in 21 cases by 2 studies [15,17]. Cold hypersensitivity (cold intolerance) was noted in eight cases from one report [17]. Persistent pain was described in eight cases within a single series [17], while intermittent pain was recorded in one case [13]. Clinodactyly was reported in eight cases by two reports [13,17]. A requirement for bone grafting was documented in six cases within one study [20], reflecting instances of comminution or delayed union. Osteomyelitis occurred in two cases [14,17], while osteolysis was described in one patient [14]. Isolated single-case reports included osteitis [11] and radial deviation and rotational malunion [13]. No major long-term morbidity related to these minor complications was reported.

Risk of Bias

Total NOS scores awarded ranged from four to six, with all studies assessed as having a moderate risk of bias (Table 2). Although the comparability was low in most studies, selection and exposure were of higher quality, indicating generally adequate cohort selection and outcome domains. The lower comparability domain scores awarded were due to the lack of control groups in the case series. Overall, all studies were of moderate quality observational evidence.

**Table 2:** Newcastle-Ottawa Scale risk of bias assessment for included studies

Author	Year	Selection	Comparability	Outcome	Total	Risk
Suzuki et al. [4]	1994	**		***	5	Moderate
De Soras et al. [11]	1997	**		***	5	Moderate
De Smet and Fabry [12]	1998	***		*	4	Moderate
Duteille et al. [13]	2003	***		***	6	Moderate
Majumder et al. [14]	2003	***		***	6	Moderate
Keramidas and Miller [15]	2005	***		***	6	Moderate
Keramidas et al. [16]	2007	***		**	5	Moderate
Debus et al. [17]	2010	***		**	5	Moderate
Finsen [18]	2010	**		***	5	Moderate
Kiral et al. [19]	2014	***		***	6	Moderate
Nanno et al. [20]	2019	***		***	6	Moderate
Kaplan et al. [21]	2024	***		***	6	Moderate

Discussion

This systematic review and meta-analysis evaluated clinical and radiographic outcomes following the management of intraarticular phalangeal fractures using the Suzuki pins and rubbers traction system. Across 12 studies (183 patients, 185 fractures), the technique demonstrated excellent fracture union rates (97.8%) and satisfactory recovery of range of motion and grip strength.

The Suzuki frame provides dynamic external fixation. Unlike static fixation, its dynamic design prevents joint stiffness and tendon adhesions, key contributors to poor recovery [22]. An analysis of 43 studies by Wang et al. demonstrated that dynamic external fixation achieves superior functional outcomes and lower complication rates than static fixation or traditional internal fixation [23]. However, it should be noted that this review included various dynamic external fixation techniques, not exclusively the Suzuki method.

The Suzuki technique demonstrated a high ROM recovery. The pooled PIPJ ROM was 89.6 degrees (95% CI 88.4 - 90.7, p<0.001), and DIPJ ROM was 66.0 degrees (95% CI 64.6 - 67.4, p<0.001), sufficient for normal hand function. Complex pilon or comminuted fractures demonstrated slightly reduced ROM and higher variability, consistent with more complex recovery processes. Studies with longer follow-ups did not report improved PIPJ or DIPJ ROM.

Grip strength recovery exceeded 70% in most studies, with modest traction periods of 4-6 weeks showing optimal outcomes. Studies with the longest follow-up reported the highest upper range of grip strengths, implying that improvements continue with rehabilitation over time, while early removal (~25 days) was linked to weaker results [17,18].

Several complications can arise in the surgical and conservative treatment of MCPJ fractures [24]. Within this review, complications were infrequent. Pinsite infections occurred in 10.3% of cases and responded well to antibiotic and hygiene management. In five cases, infection or intolerance necessitated early frame removal [13,14].

Longer traction periods appear protective against joint space narrowing [18]. There appears to be no clear relationship between joint space narrowing and age, gender or specific finger. Post-traumatic arthritis was uncommon (2.7%). Pain scores remained minimal (VAS <2), with one study accounting for most persistent pain reports--possibly a study-specific factor [17].

Excellent radiographic union rates (97.8%) confirm the reliability of dynamic external traction for maintaining alignment and promoting fracture healing. Studies using shorter traction durations (<4 weeks) reported lower union rates [13], while maintaining traction for 4-6 weeks consistently produced complete union.

Strengths and Limitations

This systematic review and meta-analysis represents the most comprehensive synthesis to date of clinical outcomes with the Suzuki system, following PRISMA 2020 standards and employing NOS risk-of-bias assessment. The pooled analysis incorporated appropriate single-arm continuous and dichotomous models, enhancing methodological robustness.

Limitations include the retrospective nature of all included studies, lack of comparator groups, variable reporting standards, and small sample sizes. The estimation of missing standard deviations introduced potential imprecision. Incomplete data on surgeon experience limited analysis. Heterogeneity in study design reporting standards, follow-up duration, and rehabilitation protocols likely contributed to variability in pooled estimates. Publication bias remains possible given the small study nature of the literature.

Despite these limitations, the overall consistency of results across diverse cohorts supported the clinical reliability of the Suzuki traction system. The technique suggests outcomes comparable to alternative fixation methods while preserving motion and minimising complications [25]. The findings provide a reference for surgical decision-making and highlight the need for prospective comparative studies with standardised outcome measures to further define its long-term functional and radiographic efficacy.

## Conclusions

Across the included studies, patient populations and fracture patterns were broadly comparable, though some variation in technique and follow-up durations was present. The majority of studies reported successful outcomes using the Suzuki pins and rubber external fixation method for intra-articular phalangeal fracture management. The results suggest that the Suzuki pins and rubber traction system used in the management of intra-articular fractures of the digits provides reliable fracture union rates (97.8%). Functional recovery was adequate; ROM at the PIPJ was 90 degrees, ROM at the DIPJ was 66 degrees, and grip strength was 81%. The overall complication rate was low, with pinsite infections at 10.3% and joint space narrowing at 5%. Shorter traction durations (<4 weeks) were associated with lower union rates, suggesting that maintaining traction for 4-6 weeks optimises bone healing. A longer traction period appears protective against joint space narrowing. Modified techniques did not compromise union or function.

The Suzuki traction system can be considered a safe, effective and minimally invasive option for managing complex intra-articular phalangeal digital fractures. Further and more robust research is necessary to support the promising outcomes suggested by this review.
